# Perception of neurology among undergraduate medical students – what can be done to counter neurophobia during clinical studies?

**DOI:** 10.1186/s12909-023-04405-y

**Published:** 2023-06-16

**Authors:** Šarūnas Jukna, Kristijonas Puteikis, Rūta Mameniškienė

**Affiliations:** 1 Faculty of Medicine, Vilnius University, Vilnius, Lithuania; 2 Center of Neurology, Faculty of Medicine, Vilnius University, Vilnius, Lithuania

**Keywords:** Neurophobia, Medical education, Neurology, Neuroanatomy, Online teaching

## Abstract

**Background and purpose:**

With a global increase in the burden of neurological diseases, the aversion towards neurology (*neurophobia*) may challenge the sufficient provision of new specialists in this field. We investigated the possible determinants of neurophobia among medical students and its influence on the intent to pursue neurology residency.

**Methods:**

From September 2021 to March 2022, an online questionnaire was distributed to medical students in Lithuania. It included questions about knowledge, confidence, interest, and teaching quality of various medical specialties (including neurology), as well as the willingness to choose neurology for residency.

**Results:**

Eight hundred fifty-two students responded to the survey (77.2% female) – they rated neurology as significantly more difficult than other medical areas and lacked confidence in assessing patients with neurological problems (*p* < 0.001). However, neurology was selected as one of the most interesting subjects and was reportedly well-taught. The prevalence of neurophobia among respondents was 58.9%. Most of them (207, 87.7%) indicated that neurology professors positively affected their outlook towards this medical specialty – such experience was associated with lower odds of neurophobia (odds ratio (OR) = 0.383, 95% confidence interval (CI) = 0.223 to 0.658). Being less neurophobic (OR = 1.785, 95% CI = 1.152 to 2.767) and having conducted neurology research (OR = 2.072, 95% CI = 1.145 to 3.747) increased the odds of a student being willing to pursue a career in neurology.

**Conclusion:**

Neurophobia was frequent among students in Lithuania and was inversely related to the positive influence by neurology professors. Together with previous research experience in the field, low levels of neurophobia were associated with the inclination to enter neurology residency.

**Supplementary Information:**

The online version contains supplementary material available at 10.1186/s12909-023-04405-y.

## Introduction

The term neurophobia was coined in 1994 by Ralph Jozefowicz to describe “a fear of the neural sciences and clinical neurology that is due to students’ inability to apply their knowledge of basic sciences to clinical situations” [1]. Later research has shown that students, trainees and practicing doctors often view neurology as a challenging field in which they lack confidence [2–4]. Neurophobia is a concerning phenomenon, as it may contribute to a shortage of neurologists at a time when brain disorders have become major contributors to disability and death worldwide [5, 6]. Limited clinical exposure, inadequate transition from preclinical studies and overwhelming learning material are cited as drivers of neurophobia, which is thought to have roots early in medical education [2, 7]. Reforming neurology courses is recommended to adapt to students’ preferences, enhance their learning, and inform students about the contemporary developments that make neurology an attractive specialty [7].

The aim of the study is to assess the prevalence and determinants of neurophobia among medical students in Lithuania.

## Methods and materials

### Study setting and questionnaire

We conducted a cross-sectional online survey by distributing virtual questionnaires through the institutional student mailing lists of the two tertiary medical education institutions in Lithuania: the Faculty of Medicine of Vilnius University (VU) and the Medical Academy of the Lithuanian University of Health Sciences (LSMU). We selected these institutions because they are the only two universities in Lithuania that offer medical studies. Both universities have similar admission processes, and future students are primarily selected based on their performance in national high school exams. While it is common to begin medical studies immediately after high school in Lithuania, studying medicine as a second degree or enrolling later in life is also possible. The study programs at both universities last for six years, and students transition to clinical modules at around year three. Both universities have major university hospitals (The Hospital of Lithuanian University of Health Sciences Kauno klinikos and Vilnius University Hospital Santaros Klinikos) that serve as clinical bases throughout the curriculum. After year six, students may apply for residency at both universities.

We also uploaded the link to the survey in closed social network groups that were accessible to students from the two study sites. In addition, we included the link in the official website of Vilnius University and weekly newsletters during the study period. Our goal was to reach all undergraduate medical students who were enrolled in years two to six of the medical curriculum at the time of study (from September 2021 to March 2022). According to government data, the largest estimate of the target population was 4152 students across the two universities [8].

Two survey forms were distributed simultaneously: one for students in years 5–6 (they either had completed the neurology course at VU or were enrolled in neurology at LSMU) and one for students in years 2–4 (no or introductory exposure to neurology). The survey was based on the neurophobia-oriented questionnaire by Schon et al., which used Likert scales from one to five to evaluate knowledge, difficulty, confidence, and interest in different medical specialties. Additional questions about the students’ vision of neurology and its teaching were included in the survey for students in years 5–6 (each item is presented in Supplementary Box 1) [4]. The survey was created ad hoc by study authors Š.J. (medical student), R.M. (professor in neurology) after a review of the literature and further discussion of 1) possible preconceived opinions about neurology in general, 2) possible factors making neurology difficult and 3) determinants of the quality of the neurology course. Additional items in the survey included demographic information, the way of how neurology was taught (e.g., in-person or online), the students’ score in neurology, their previous research experience in neurology or neurosciences, and their willingness to pursue neurology residency after undergraduate medical studies.

The questionnaire for students in years 5–6 included items about the teaching of neurology and the student experience during the course, whereas the questionnaire for students in years 2–4 was identical but did not include these items. First-year students were excluded due to their lack of study experience at the time of the survey. Open-source information indicates that students at both universities receive 9–10 European Credit Transfer and Accumulation System (ECTS) credits for completing their neurology training [9, 10].

Prior to distribution, the questionnaire was re-evaluated for face validity that may be understood as the “degree that respondents or end users [or lay persons] judge that the items of an assessment instrument are appropriate to the targeted construct and assessment objectives” [11, 12]. Because of the straightforward nature of the questions and their adaptation to the grammatical structure of the Lithuanian language, they were considered to be easily comprehensible, relevant, and of appropriate length. As each question was scored independently and did not form a scale, this evaluation was deemed sufficient to confirm the validity of the questionnaire. Students were given an unlimited amount of time to complete the questionnaire before submitting it and were not provided any incentives to do so.

### Ethics

The anonymous online survey was not subject to ethical approval under Lithuanian law, as it does not meet the legal definition of a biomedical study. Willing participants expressed their consent to provide their views by voluntarily entering and completing the survey. Throughout the survey process, the researchers did not have access to any identifiable information and all responses remained anonymous.

### Statistical analysis

A Wilcoxon signed-ranks test was applied to determine the difference between answers to Likert scale questions in neurology as opposed to other medical specialties. Neurophobia was defined and calculated according to Kam et al.: scores of items relating to difficulty (item “How would you evaluate neurology in terms difficulty”) and confidence (item “How would you evaluate your confidence and knowledge when examining a patient and diagnosing disorders related to neurology”) in neurology were added. This measure varied from scores of 2 to 10 and neurophobia was defined as a score of ≤ 4 (i.e., low confidence and high difficulty) [3]. Stepwise binary regression models were created to investigate the possible determinants of two endpoints – neurophobia and the willingness to enter neurology residency.

## Results

The survey was completed by 852 students (658 (77.2%) female, 236 (27.7%) were in years 5–6 and had already been exposed to the course of clinical neurology), their general characteristics are presented in Table 1. The response rate was estimated at 852/4152, or 20.5% without acknowledging exclusion of 1^st^ year students [8].

**Table 1:** General characteristics of the study sample

Characteristic	Frequency (%)
**Sex**	
Male	191 (22.4)
Female	658 (77.2)
Non-binary	3 (0.4)
**University**	
Vilnius University	437 (51.3)
Lithuanian University of Health Sciences	415 (48.7)
**Study year**	
2	197 (23.1)
3	225 (26.4)
4	194 (22.8)
5	121 (14.2)
6	115 (13.5)
**Has close ones (e.g., family, friends, neighbors) with neurological disorders**	292 (34.3)
**Cared for someone close with a neurological disorder**	198 (23.2)

**Fig. 1:**
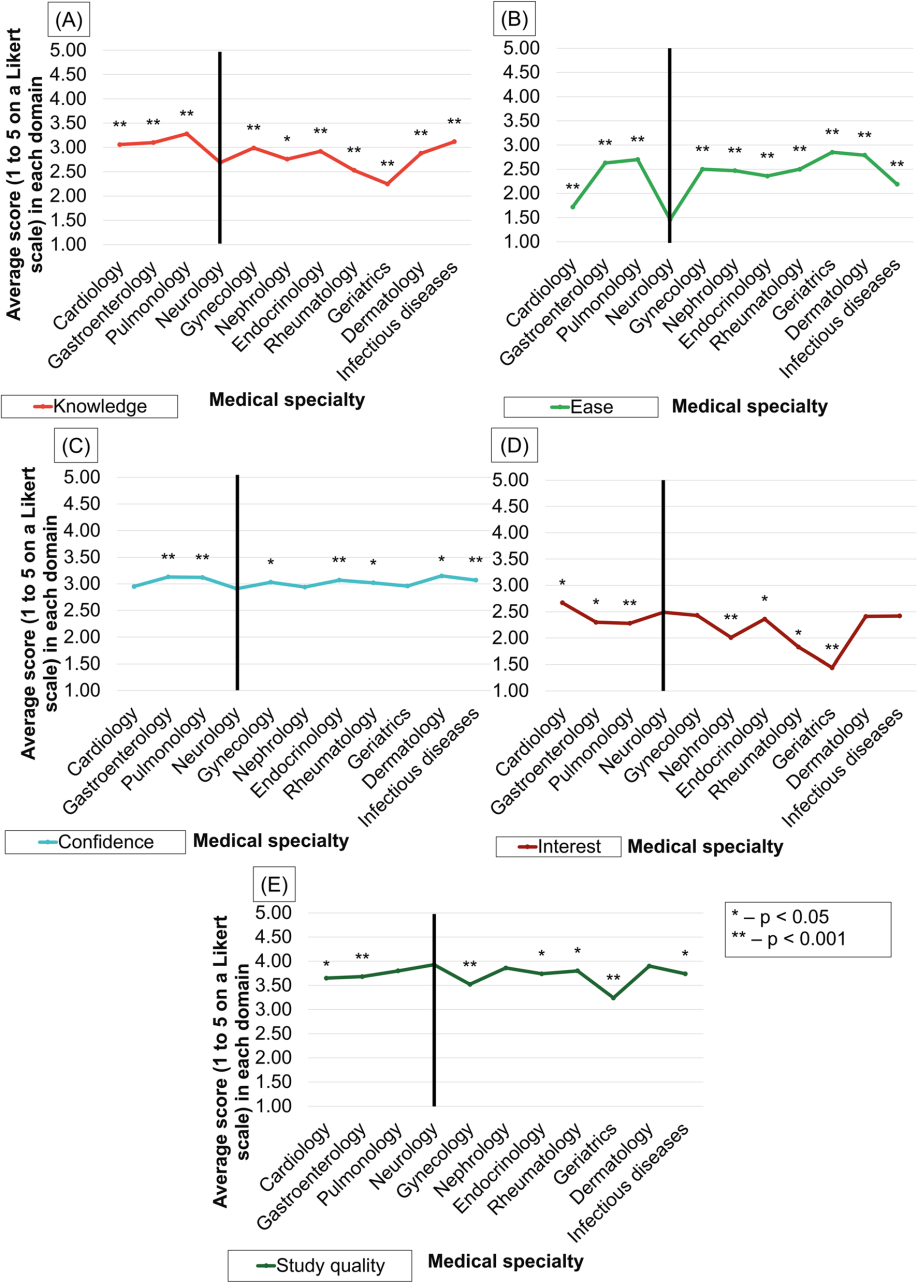
Average ratings of knowledge (**A**), ease (**B**), confidence (**C**), interest (**D**), and study quality (**E**) in different medical specialties. Asterisks mark statistically significant difference from scores attributed to neurology

While neurology was perceived to be more difficult than other specialties and respondents were usually less confident and knowledgeable in this field, it was seen as one of the most interesting specialties (Fig. 1).

**Fig. 2:**
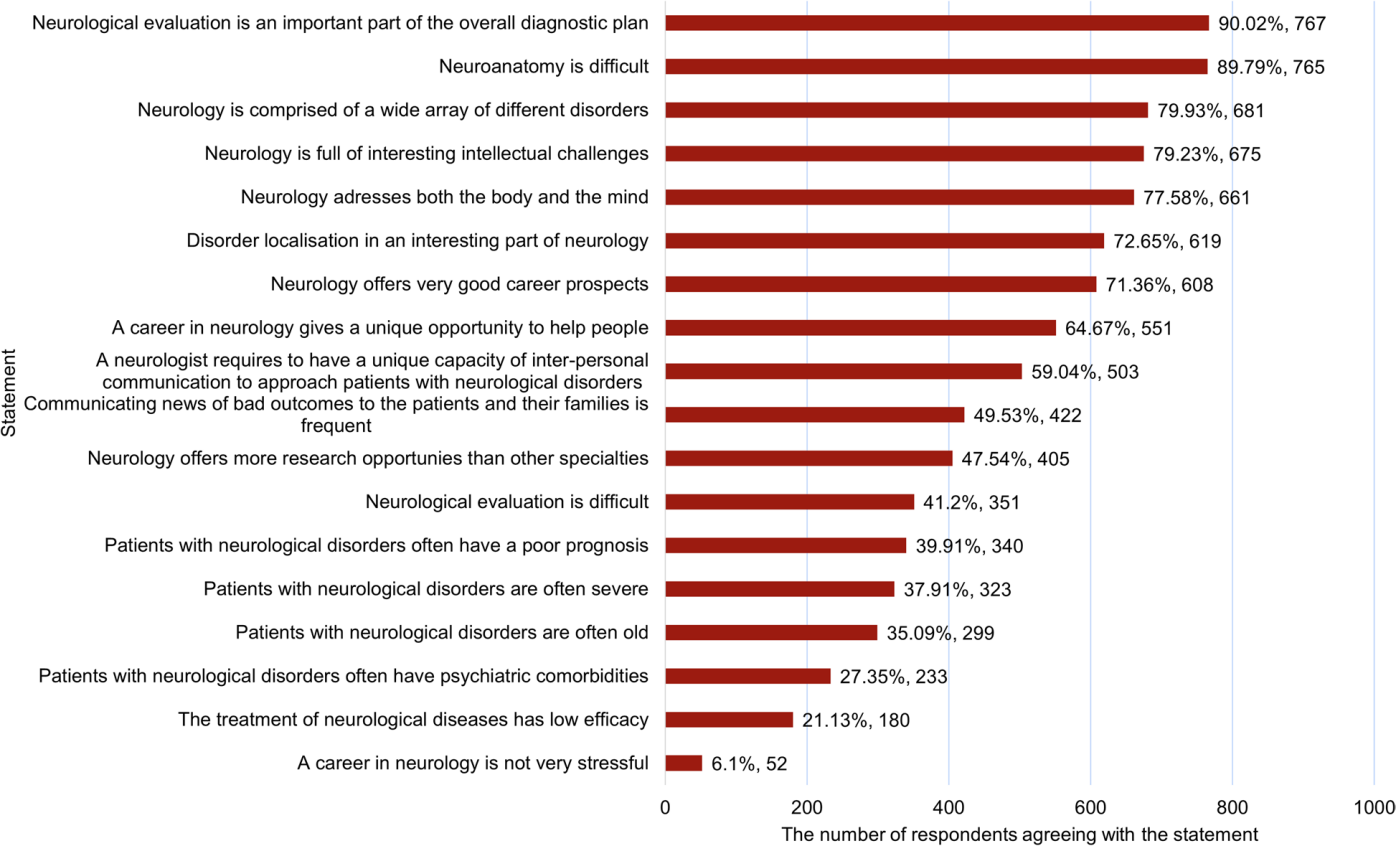
Student agreement with different statements about neurology

Students in years 5–6 indicated the quality of the course to be one of the best among all areas (alongside dermatology, nephrology, and pulmonology). Students perceived neurology as a heterogeneous (agreed by 681, 79.9% respondents) and intellectually challenging field (675, 79.2%) that has good career prospects (608, 71.4%), Fig. 2. Only a minority of students perceive neurology to be a relaxed specialty (52, 6.1%) and nearly forty percent associate it with the care of elderly, severe patients and those having poor prognosis.

The overall prevalence of neurophobia was 58.9% (427 of 725 respondents who evaluated both the difficulty of neurology and their confidence in this area of medicine) and was similar among students of different study years (52.5, 63.2, 62.0, 54.7 and 59.6% in years two to six, respectively, χ^2^ = 5.33, *p* = 0.255).

Most students in years 5–6 of their undergraduate medical studies received neurology education through a combination of online and in-person teaching (97, 41.1%), whereas 84 (35.6%) received courses solely online and 55 (23.3%) only in person. Neurophobia was evaluated to be more present among those who studied neurology only online (median = 4, IQR = 3–5) or in person (median = 4, IQR = 3–5) in comparison to students who were taught in a mixed method (median = 5, IQR = 4–6, H(2) = 8.79, *p* = 0.012, a higher score means a more positive outlook towards neurology). Students who had experienced both in person and online teaching performed better on their neurology tests (H(2) = 14.35, *p* = 0.001).

**Fig. 3:**
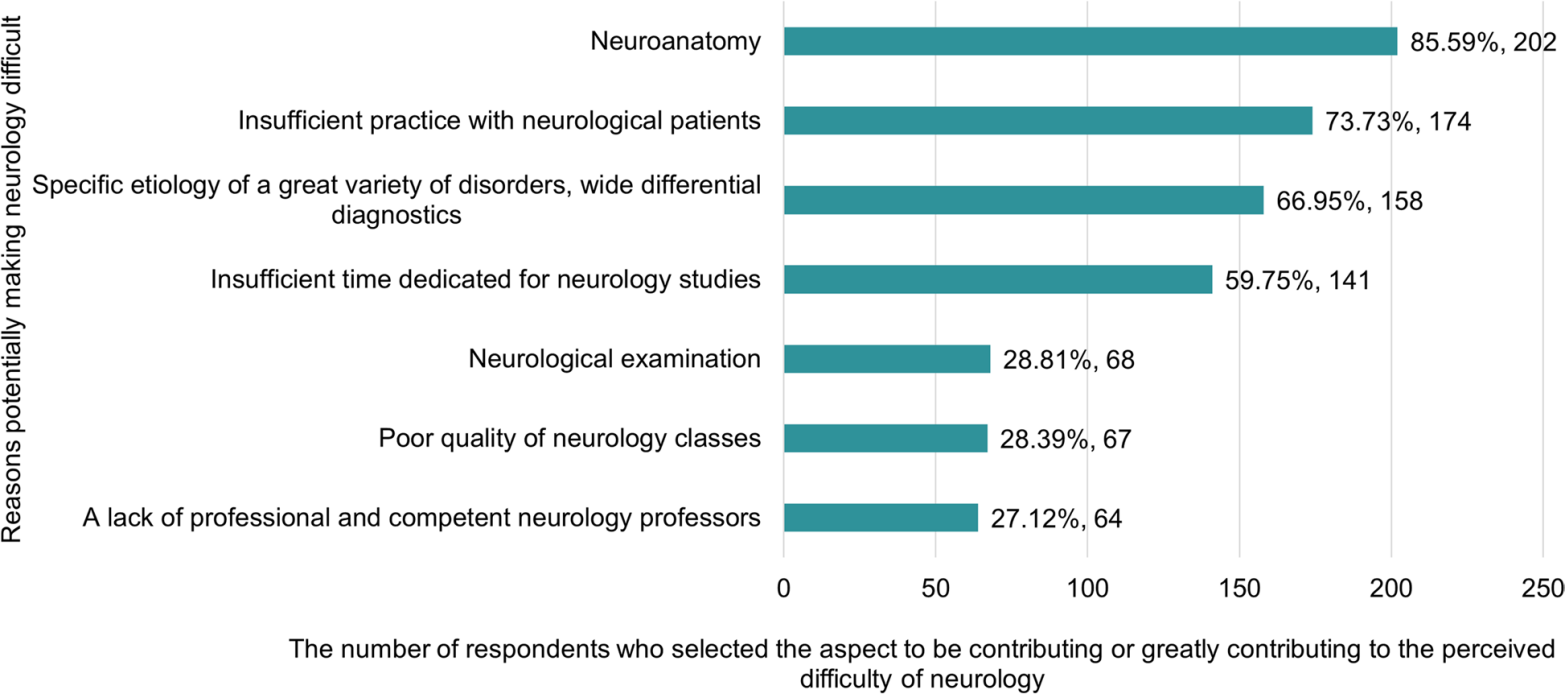
Reasons making neurology a difficult medical area (responses by medical students in years 5–6)

**Fig. 4:**
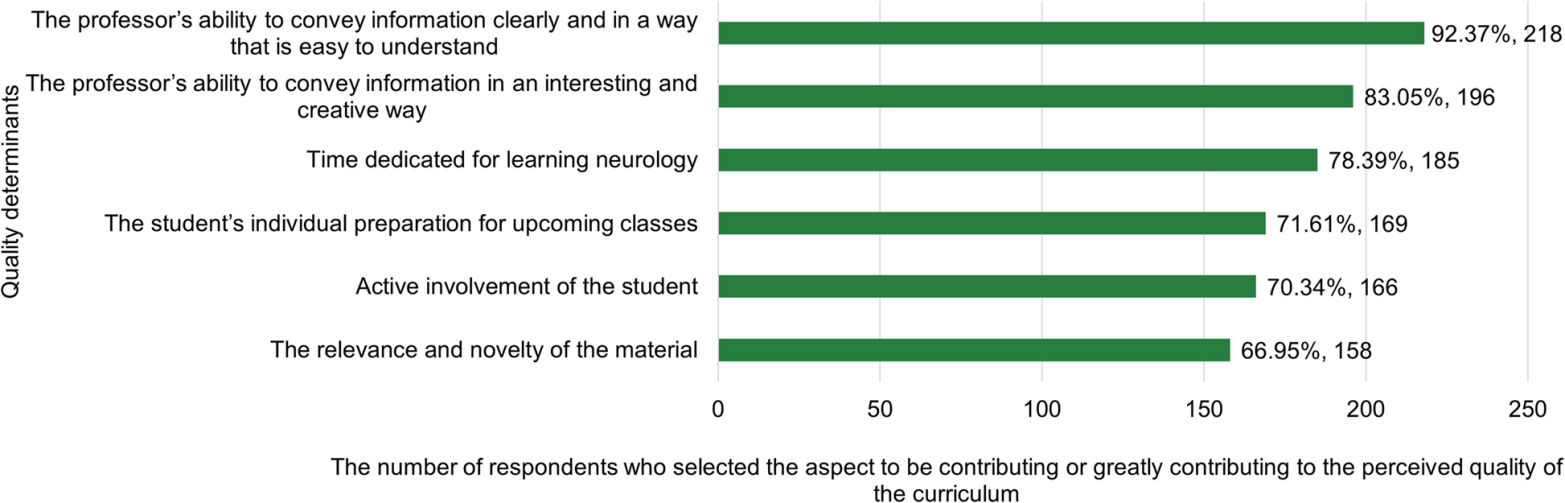
Determinants of the quality of the neurology course ((responses by medical students in years 5-6)

Neuroanatomy (202, 85.6%), insufficient practical skills (174, 73.7%) or time to learn study material (141, 59.8%) as well as the need to consider a wide array of etiological factors and syndromes in differential diagnostics (158, 67.0%) were selected as the major causes making neurology difficult (Fig. 3). According to the respondents, clear (218, 92.4%) or interesting and creative (196, 83.1%) teaching was the main determinant of the course’s quality (Fig. 4). About a third of students (77, 32.6%) believed that the time dedicated to the course was sufficient to cover all relevant topics. Most students reported receiving good or very good teaching (215, 91.1%) and indicated that their professors had a positive impact on their experience of neurology (207, 87.7%) or were good examples of professionalism (194, 82.2%).

There were 74 (8.7%) respondents (among them, 59 (79.7%) studied in years 2–4) seeing neurology as a likely or definitive career choice. A binary regression model showed that a positive influence of professors on the student experience of neurology was associated with lower odds of neurophobia (Table 2A). The latter was related to the intention of selecting neurology as a career path (Table 2B).

**Table 2:** Stepwise binary regression models with neurophobia and the intention to choose neurology after medical studies as dependent variables

Variable	Beta coefficient	Wald	*P* value	Odds ratio	95% Confidence interval
(A) Dependent variable: Presence of neurophobia (*n* = 203), Nagelkerke R^2^ = 0.089, model χ^2^ = 13.953, *p* < 0.001					
Positive teaching impact on experience in neurology	-0.961	12.075	0.001	0.383	0.223 to 0.658
Constant	3.138	13.289	< 0.001	23.051	
(B) Dependent variable: Intention to select neurology as a career path (*n* = 203), Nagelkerke R^2^ = 0.173, model χ^2^ = 14.940, *p* = 0.001					
Neurophobia score	0.580	6.722	0.010	1.785	1.152 to 2.767
Research experience in neurology	0.728	5.802	0.016	2.072	1.145 to 3.747
Constant	-7.113	24.123	< 0.001	0.001	

Excluded variables: previous research experience in neurology (part A only), positive teaching impact on experience in neurology (part B only), reported sufficient time to learn the material of the course, teaching quality, the professionalism of professors giving the course, study method (online, in person or mixed), sex, exposure to close relatives with neurological disorders, previous care for individuals with neurological disorder

## Discussion

The present study aimed to assess the prevalence of neurophobia and its determinants among undergraduate medical students in Lithuania. The survey results confirmed some previous findings about neurophobia – students perceive neurology as one of the most difficult subjects in medicine and usually do not feel confident to assess and diagnose patients with neurological problems [2–4]. The prevalence of neurophobia in our study was somewhat higher than in the original study by Kam et al. (47.5%) in which the phenomenon was quantified and defined as a combination of low confidence in neurology and perceived high difficulty of this medical area [3]. Although the exact prevalence of neurophobia may depend on the site of study as well as the respondent population (e.g., female predominance among respondents), our data further confirm that the aversion to neurology may be found in around half of all medical students [1]. Furthermore, we found that the levels of neurophobia remain relatively stable throughout the medical curriculum and may depend on preconceptions about neurology before entering medical school, as well as early experiences during the first year of preclinical studies [13].

While judging neurology as a difficult field, students found it to be one of the most interesting medical areas and reported high quality of teaching during the respective course. We believe this represents the ambivalence that students face approaching neurology – they may find this field attractive but remain overwhelmed by its complexity even despite adequate teaching (e.g., focused on frequent and the most relevant conditions) [2]. For instance, some features inherent to neurology (e.g., heterogeneity of neurological disorders and a prerequisite of understanding neuroanatomy) combined with limited time dedicated to the course remained important contributors of the perceived complexity of this area of medicine. They may potentially be countered by immersive neuroanatomy teaching, gamification of learning techniques as well as extracurricular initiatives helping familiarize with the rich variety of subfields within neurological sciences [14–17]. Our data suggests that students prioritize clear and creative ways of presenting information over the relevance of the material itself or even their active involvement during the course. Recent reports have also indicated positive effects of teaching interventions aimed at easing the delivery of information, such as roleplaying of semiology and neuroanatomy teaching in virtual reality [15, 17].

The COVID-19 pandemic hindered in-person learning but also presented an opportunity to integrate virtual teaching into medical studies. Thus, we sought to provide evidence of how different aspects of neurology teaching influence the level of neurophobia. It was shown that a mixed in-person and online format of teaching that emerged during the COVID-19 pandemic may be associated with lower levels of neurophobia and superior student performance during testing. We believe this is a novel finding suggesting that neither the reliance on physical student presence nor a full transition to virtual learning is beneficial in tackling neurophobia. Therefore, integrating modern teaching techniques within courses that rely on conventional instruction may be the optimal choice for intrinsically complex material such as neuroanatomy [18, 19]. For example, the eNEUROANAT-CF (e-neuroanatomy learning conceptual framework) has several theoretical underpinnings that facilitate the learning of neuroanatomy, such as avoidance of information overload, learning style, contexualization, motivation, social learning, reflective practice and feedback and active learning [20]. With the inevitable advances of technological teaching tools and their accessibility, our results suggest that their integration within the in-person course of clinical neurology should be further investigated.

Despite the significance of the methods and quality of teaching neurology, in our study neurophobia was most strongly related to the overall subjective perception of how professors influenced the student experience in the course (for instance, teaching quality or professors’ professionalism were not included as statistically significant variables in the regression model). This suggests that individual factors that are difficult to define may influence neurophobia. It remains unclear, however, whether the student experience is largely predefined by the students’ characteristics or may be modified during the neurology course. Even if the latter was true, only around one in every eight students reported that neurology professors swayed them away from neurology – this results in little room for improvement and further affirms the notion that preclinical studies represent “a therapeutic window for neurophobia” [17]. As neurology professors may not give classes during preclinical years, other opportunities for addressing neurophobia should be explored. One possibility is improving collaboration between anatomy professors and clinical specialists to enhance teaching neuroanatomy to younger students. Although learning neuroanatomy is considered a major contributor to neurophobia, evidence-based approaches to improve neuroanatomy teaching appear to be scarce [15, 19]. Therefore, immersive neuroanatomy teaching with a focus on the clinical significance of various structures should be further studied for its applicability and effectiveness [21]. Based on our findings, one of the ways to counter neurophobia in later years of the medical curriculum may be the involvement of undergraduate students in research. We cannot speculate about the directionality of the detected association between previous experience in neurology research and the willingness to pursue a career in neurology. However, undergraduate students are usually interested in participating in scientific research that might help them overcome the perceived difficulty of neurology and choose it as their career path [22]. Inviting medical students to local or international conferences related to neurology or neurosciences may a potential starting point to spark interest in these fields and increase student participation in research activities that can supplement their medical curriculum.

The limitations of our study include its single-country design and results being acquired through a virtual and anonymous survey form, which may lead to respondent bias and non-participation of students having little or no interest in reporting their views on neurology or evaluating their neurology course. For instance, respondents in our sample were predominantly female. While we believe this is generally representative of the population of medical students in Lithuania, our results could be less applicable in settings with a higher proportion of male students. Further, the study was done in two universities that both autonomously decide on how the preclinical neurosciences and clinical neurology are taught throughout the medical curriculum. This could lead to affiliation-related heterogeneity within our sample. This limitation was countered by focusing the questionnaire on universally understandable and abstract concepts that are applicable to students from both institutions.

## Conclusions

The results of our survey confirm that neurophobia is a common phenomenon among medical students from the early years of their studies. We found that a positive influence from neurology professors can potentially help decrease the level of neurophobia among students in their clinical years. Although neurophobia may discourage some students from pursuing a neurology residency, we also discovered that exposure to neurology research during their undergraduate studies can positively impact their inclination towards this career path.

## Supplementary Information

**Additional file 1:** **Supplementary Box 1.** Questions included in the questionnaire that was used in the study (translated from Lithuanian).

## Data Availability

The datasets used and/or analyzed during the current study available from the corresponding author on reasonable request.
