# Supplementary material for: Perception of neurology among undergraduate medical students – what can be done to counter neurophobia during clinical studies?
Source: BMC Med Educ. 2023 Jun 16;23:447. doi: 10.1186/s12909-023-04405-y (PMC10276433; doi:10.1186/s12909-023-04405-y)
Supplement: Supplementary file 1 — Additional file 1: Supplementary Box 1. Questions included in the questionnaire that was used in the study (translated from Lithuanian). [file 12909_2023_4405_MOESM1_ESM.docx]

**Supplementary Box 1.** Questions included in the questionnaire that was used in the study (translated from Lithuanian). (*) – only completed by students in years 5-6 who had already been exposed to undergraduate neurology training.

| - Demographic information (sex, university, year of studies) |
| --- |
| - What was the type of neurology studies you experienced: in-person, online or mixed? (because of the COVID-19 pandemic in 2020-2021, some students had been enrolled in an fully online neurology course and some had classes in person or in a mixed format)* |
| - Do you have close ones (e.g., family, friends, neigbours) with a neurological disorder? |
| - Did you care for someone close to you with a neurological disorder? |
| - How would you evaluate your **knowledge** in the following medical areas: cardiology, gastroenterology, pulmonology, neurology, gynecology, nephrology, endocrinology, rheumatology, geriatric medicine, dermatovenerology, infectious diseases (scale 1 [very bad] to 5 [very good])? |
| - How would you evaluate the following medical areas in terms of their **difficulty** (aforementioned areas, scale 1 [very difficult] to 5 [very easy])? |
| - How would you evaluate **your confidence and knowledge when examining a patient** and diagnosing disorders related to the following medical areas (aforementioned areas, scale 1 [very uneasy] to 5 [very confident])? |
| - How would you evaluate your **current interest** in the following medical areas (aforementioned areas, scale 1 [not interested] to 5 [very interested])? |
| - How would you evaluate the **study quality** the following medical areas (aforementioned areas, scale 1 [very poor] to 5 [very good])?* |
| - Agreement with statements (scale 1 [completely disagree] to 5 [completely agree]) regarding neurology: |
| - - Patients with neurological disorders often have a poor prognosis |
| - - The treatment of neurological diseases has low efficacy |
| - - Patients with neurological disorders are often old |
| - - Neuroanatomy is difficult |
| - - Communicating news of bad outcomes to the patients and their families is frequent |
| - - Neurological evaluation is difficult |
| - - Patients with neurological disorders often have psychiatric comorbidities |
| - - Patients with neurological disorders are often severe |
| - - A career in neurology gives a unique opportunity to help people |
| - - Neurological evaluation is an important part of the overall diagnostic plan |
| - - Neurology offers more research opportunies than other specialties |
| - - Disorder localisation in an interesting part of neurology |
| - - Neurology is full of interesting intellectual challenges |
| - - A neurologist requires to have a unique capacity of inter-personal communication to approach patients with neurological disorders |
| - - Neurology is comprised of a wide array of different disorders |
| - - A career in neurology is not very stressful |
| - - Neurology adresses both the body and the mind |
| - - Neurology offers very good career prospects |
| - Did you have an opportunity to work on a research project in neurology or neuroscience? |
| - Neurology may appear difficult because of various reasons. Please evaluate how the listed items contribute to the difficulty of neurology (scale 1 [does not contribute at all] to 5 [contributes a lot]):* |
| - - Neuroanatomy |
| - - Neurological examination |
| - - Poor quality of neurology classes |
| - - Insufficient practice with neurological patients |
| - - Insufficient time dedicated for neurology studies |
| - - Specific etiology of a great variety of disorders, wide differential diagnostics |
| - - A lack of professional and competent neurology professors |
| - What determines the quality of the neurology course (scale 1 [does not influence quality] to 5 [greatly influences quality])?* |
| - - Time dedicated for learning neurology |
| - - The professor’s ability to convey information in an interesting and creative way |
| - - Active involvement of the student |
| - - The professor’s ability to convey information clearly and in a way that is easy to understand |
| - - The student’s individual preparation for upcoming classes |
| - - The relevance and novelty of the material |
| - Did your professor(s) have sufficient time to cover all the planned topics relating to neurology?* |
| - Please evaluate the quality of your professor(s) teaching of neurology (scale 1 [very poor] to 4 [very good])* |
| - How did your professor(s) of neurology affect your overall experience in this subject (scale 1 [affected in a very negative way] to 4 [affected in a very positive way])?* |
| - Were your professors in neurology good examples of professionalism in both theorethical and practical contexts of neurology studies?* |
| - How likely are you to pursuit a career in neurology? |
| - What was your grade in neurology?* |
